# Comparison on Tensile Characteristics of Plain C–Mn Steel with Ultrafine Grained Ferrite/Cementite Microstructure and Coarse Grained Ferrite/Pearlite Microstructure

**DOI:** 10.3390/ma14092309

**Published:** 2021-04-29

**Authors:** Yan Tian, Mingchun Zhao, Wenjian Liu, Jimou Zhang, Min Zhang, Hongying Li, Dengfeng Yin, Andrej Atrens

**Affiliations:** 1 School of Materials Science and Engineering, Central South University, Changsha 410083, China; 163111055@csu.edu.cn (Y.T.); mczhao@csu.edu.cn (M.Z.); 0701130416@csu.edu.cn (W.L.); lhying@csu.edu.cn (H.L.); 2 Xiangtan Iron & Steel Co., Ltd. of Hunan Valin, Xiangtan 411101, China; jmzhang@mail.hnxg.com.cn; 3 Hengyang Valin Steel Tube Co., Ltd., Hengyang 421001, China; mzhang@hysteeltube.com; 4 School of Mechanical and Mining Engineering, University of Queensland, Brisbane, QLD 4072, Australia; andrejs.atrens@uq.edu.au

**Keywords:** plain C–Mn steel, tensile property, strength, ductility, grain size

## Abstract

This work investigated the tensile characteristics of plain C–Mn steel with an ultrafine grained ferrite/cementite (UGF/C) microstructure and coarse-grained ferrite/pearlite (CGF/P) microstructure. The tensile tests were performed at temperatures between 77 K and 323 K. The lower yield and the ultimate tensile strengths were significantly increased when the microstructure was changed from the CGF/P to the UGF/C microstructures, but the total elongation and the uniform elongation decreased. A microstructural change from the CGF/P microstructure to the UGF/C microstructure had an influence on the athermal component of the lower yield and the ultimate tensile strengths but not on the thermal component. The UGF/C microstructure with a higher carbon content provided a higher strength without losing ductility because cementite particles restrained necking.

## 1. Introduction

Plain C–Mn steels containing no costly alloying elements are economical structural materials. The conventional microstructure of plain C–Mn steels consists of coarse ferrite grains and pearlite colonies, i.e., coarse ferrite grains and alternate lamellae of cementite and ferrite (hereafter, the CGF/P microstructure), which have been in commercial production for centuries. In recent years, the microstructure consisting of ultrafine ferrite grains and nanosized globular cementite particles (hereafter, the UGF/C microstructure) was also developed in plain C–Mn steels by means of the heavy deformation of the CGF/P microstructure [1,2,3]. The UGF/C microstructure is more desirable for strengthening steel due to the ultra-refinement of the ferrite grains and the dispersion strengthening caused by the nanosized cementite particles. So far, the UGF/C microstructure has been directly applied in research and practical production, which does not require annealing. For engineering applications, steels must meet the requirements of strength and ductility, which are measured in a tensile test [4,5]. Therefore, these mechanical properties are an important performance target for steels. However, there has been little systematic investigation on the characteristic tensile properties of plain C–Mn steels with the UGF/C microstructure, and, specifically, there has been no explicit comparison on the difference in the tensile characteristics of plain C–Mn steels with the UGF/C microstructure and the CGF/P microstructure. For example, it is interesting that what is different in terms of the strength, elongation and tensile fracture of the microstructure in plain C–Mn steels changes from the CGF/P microstructure to the UGF/C microstructure. Another interesting element is how to make ultrafine grains have a high strength without losing ductility. This work investigated the tensile characteristics of plain C–Mn steel with a UGF/C microstructure and a CGF/P microstructure, and aimed to obtain a high strength without a loss of ductility for plain C–Mn steels with a UGF/C microstructure.

## 2. Materials and Methods

The chemical compositions (wt.%) of plain C–Mn steels are identified as steel A: 0.15 C, 1.51 Mn, 0.31 Si, 0.01 Al, 0.001 P, 0.002 S, 0.001 O, 0.001 N; and steel B: 0.45 C, 1.51 Mn, 0.31 Si, 0.02 Al, 0.001 P, 0.002 S, 0.001 O, 0.001 N. The difference in their chemical compositions was mainly in the carbon content (0.15 wt.% and 0.45 wt.%, respectively), with little difference in other elements. The main research work was focused on steel A, with steel B only used as the controls to investigate the influence of the carbon content in UGF/C microstructures.

The ingots were melted at a laboratory scale in a vacuum and homogenized at 1473 K for 60 min. The UGF/C microstructure and the CGF/P microstructure were obtained from the ingots as follows. For the UGF/C microstructure, the ingots were hot-forged into rods with a 115-mm diameter. The obtained hot-forged rods were reheated at 1173 K for 60 min, and were subsequently caliber-warm-rolled [6]: i.e., caliber-groove-rolled to 79-mm square rods at 1073 K, to 24-mm square rods at 823 K, and finally to 17-mm square rods at 723 K, followed by water cooling. For the CGF/P microstructure, the ingots were hot-rolled from 40-mm- to 20-mm-thickness plates at 1273 K, followed by air cooling.

The microstructures of the specimens were characterized in the transverse sections using scanning electron microscopy (SEM), electron back scattered diffraction (EBSD) and transmission electron microscopy (TEM). Samples for SEM were mechanically grinded, polished and etched in a 2% natal solution. Samples for EBSD and TEM were mechanically and electrochemically polished using a (10 vol% perchloric acid + 90 vol% acetic acid) solution at 40 V. The EBSD data were measured using a field emission SEM (LEO-1550 Schottky, Carl–Zeiss, Jena, Germany) equipped with an orientation imaging microscope (OIM) system in a trademark of TexSEM Laboratories, Inc., Provo, UT, USA with a ~30-nm spot size at 25 kV. For the TEM observation that was conducted using a JEM-2000FXII microscope (Tokyo, Japan) at 200 kV, 0.2-mm-thick thin-slice samples were first mechanically ground to ~50 μm, and foil samples were obtained by electrochemical polishing into the Φ3-mm discs. The EBSD scan step was 0.1 μm.

Cylindrical tensile test pieces, with a length parallel to the rolling direction, were machined from the rolled rods or plates with a 3.5-mm gage diameter and a 25-mm gage length. Tensile tests were conducted using a 0.5-mm/min cross-head speed at 323 K, 293 K (room temperature), 210 K and 77 K, respectively. The samples were stayed in the set cryogenic temperature for 30 min in the cryogenic tank before testing. 210 K was obtained by refrigerating. 77 K was obtained directly by liquid nitrogen.

## 3. Results and Discussion

**Figure 1:**
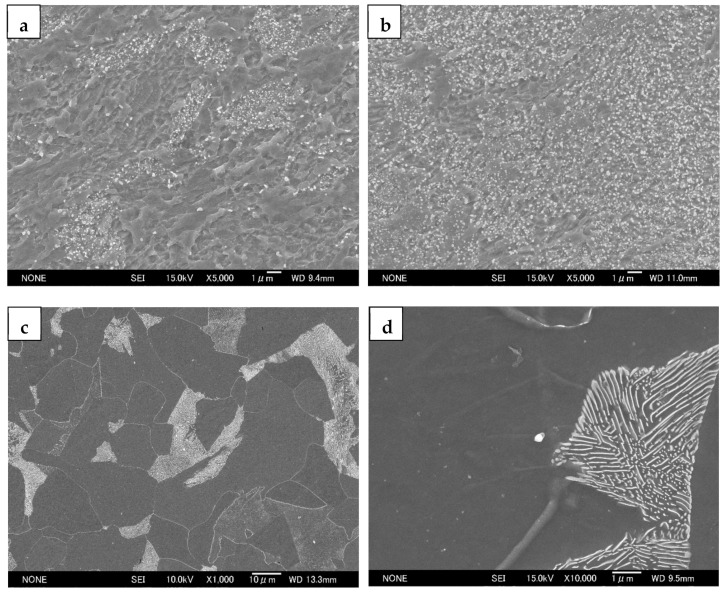
SEM microstructures for (**a**) the UGF/C microstructure in steel A, (**b**) the UGF/C microstructure in steel B, (**c**) the CGF/P microstructure in steel A and (**d**) the structural features of pearlite.

Figure 1 depicts the SEM characteristics of the UGF/C microstructure and the CGF/P microstructure. The UGF/C microstructures (Figure 1a,b) have an ultrafine grained ferrite matrix containing nanosized (smaller than 100 nm) globular cementite particles. The detailed characteristics of the UGF/C microstructures were documented previously [2]. The carbon content increase from 0.15 wt.% (steel A) to 0.45 wt.% (steel B) caused a slight decrease in the average ferrite grain size and a much higher volume fraction of cementite particles. The cementite particles presented heterogeneous dense distributions at some specific sites in steel A, while they were more homogeneously distributed in steel B. The characteristic cementite distribution was attributed primarily to the original ferrite-pearlite structure [2,7]. A higher carbon content in steel resulted in a higher volume fraction of pearlite in the original microstructure after the phase transformation, which facilitated a more homogeneous cementite distribution during caliber warm rolling because the pearlitic lamellae broke into cementite particles that localized within the pearlite colonies. The CGF/P microstructure (Figure 1c) contained ferrite and pearlite, with an average ferrite grain size of 20 μm. The higher-magnification SEM micrograph (Figure 1d) revealed structural features of pearlite, showing a typical lamellar arrangement with alternate thin ferrite and cementite layers.

**Figure 2:**
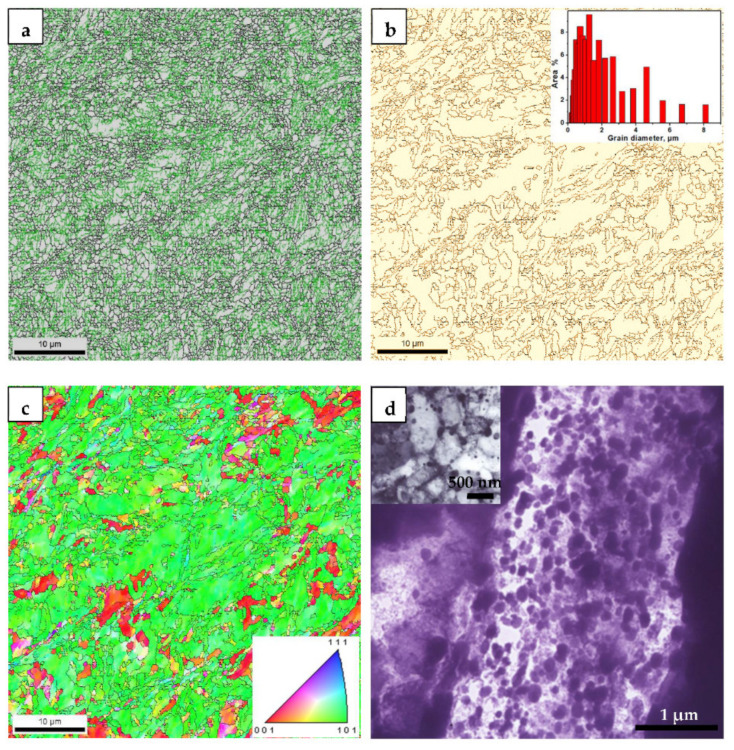
(**a**) EBSD-characterized grain boundaries, (**b**) High-angle grain boundaries and grain size distribution, (**c**) IPF map and (**d**) TEM micrographs of the UGF/C microstructure in steel A.

Figure 2a shows the EBSD-characterized grain boundaries of the UGF/C microstructures in steel A. High-angle grain boundaries (i.e., a misorientation higher than 15°) were depicted by black lines and low-angle grain boundaries (i.e., a misorientation from 1° to 15°) were depicted by green lines. There were essentially no grain boundaries with a misorientation under 1°. High-angle grain boundaries were accompanied by a large number of intragranular low-angle grain boundaries. In other words, the low-angle grain boundaries, i.e., the sub-grains, were distributed entirely in the UGF/C microstructures. This clearly indicated that the UGF/C microstructures had experienced heavy deformation [8,9] during the caliber warm rolling. For the deformed UGF/C microstructures, the grains with low-angle grain boundaries are not, strictly speaking, grains. Usually, high-angle grain boundaries are taken as the grain size in the UGF/C microstructure. Figure 2b shows the EBSD-characterized grain boundaries with a misorientation of more than 15° for the UGF/C microstructures in steel A. The grain size distribution corresponding to the grains manifested by the boundaries with a misorientation of more than 15° is also embedded in Figure 2b. The average ferrite grain size is less than 1 μm. Figure 2c shows the IPF map of the UGF/C microstructures in steel A. Most of the grain orientation was concentrated in the (101) direction, but some was distributed in the (001) direction. Figure 2d shows the TEM micrographs of the UGF/C microstructures in steel A, in which the microstructure was mainly composed of ultrafine grained ferrite and nanosized cementite particles that were primarily attributed to the broken original microstructures, i.e., the ferrite-pearlite structure, during the caliber warm rolling. These microstructural characteristics substantiated that the UGF/C microstructure was different from the conventional CGF/P microstructure.

**Figure 3:**
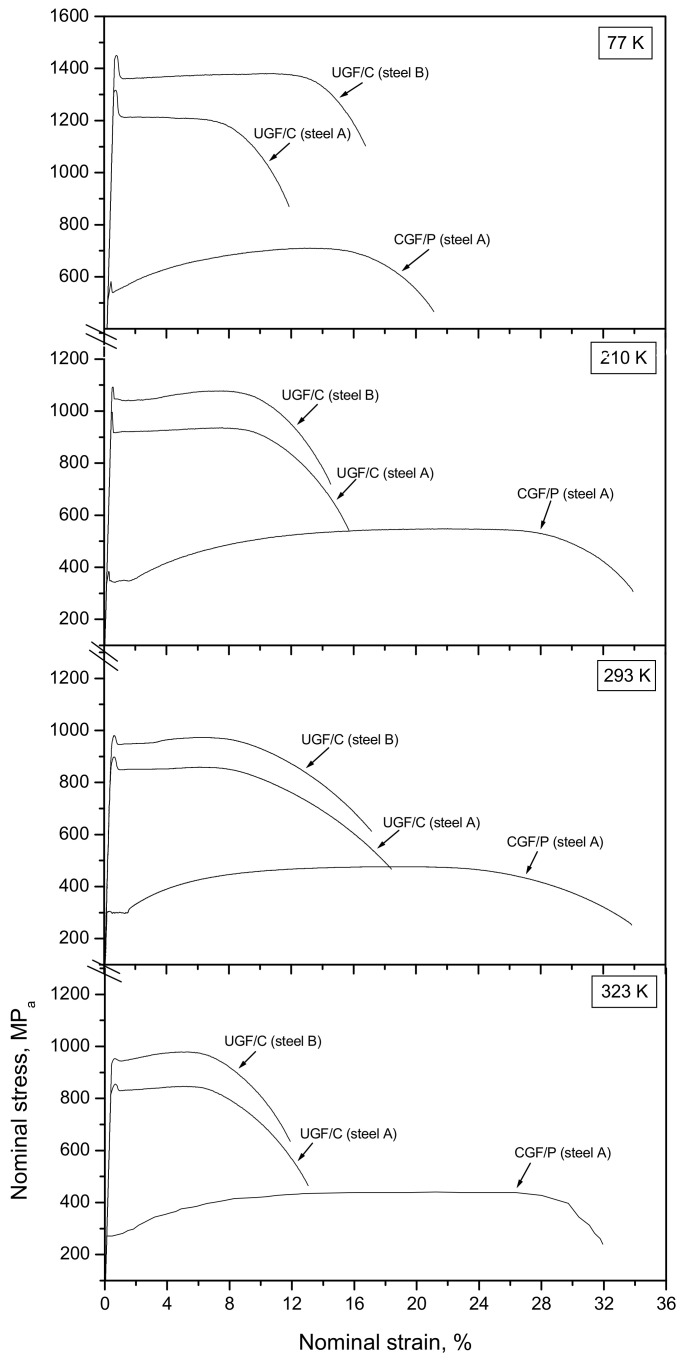
Nominal stress-strain curves at different tensile temperatures.

**Figure 4:**
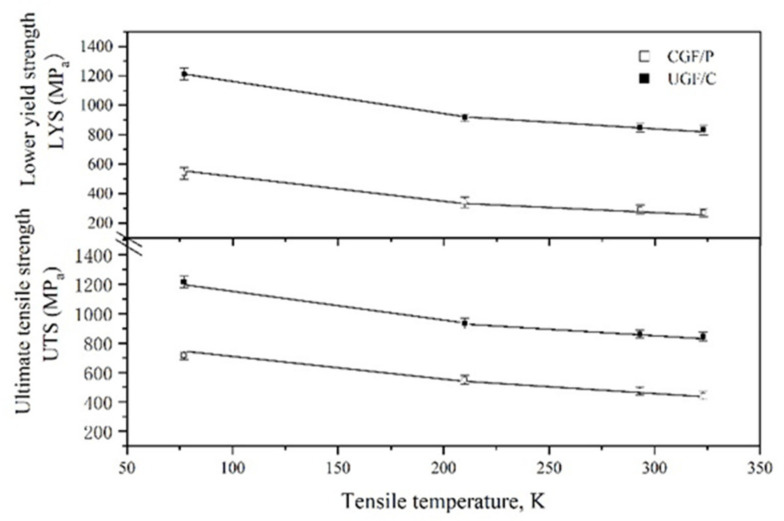
Lower yield strength and ultimate tensile strength change with the temperature for every microstructure in steel A.

Figure 3 shows the nominal stress-strain curves at different temperatures, depicting different tensile characteristics for each microstructure. The UGF/C microstructures show discontinuous yielding followed by a considerable uniform elongation. At each temperature, the total elongation (TEL), the uniform elongation (UEL), the lower yield stress (LYS) and the ultimate tensile stress (UTS) clearly increased when the carbon content increased from 0.15 wt.% (steel A) to 0.45 wt.% (steel B). TEL is the elongation from the beginning of the deformation to the final fracture, and UEL is the elongation from the beginning of the deformation to the occurrence of necking, i.e., the maximum power elongation. The LYS/UTS ratio was approximately 1. This high ratio means a low deformation resistance from the yield to the plastic instability, which was also observed in some other ultrafine grained metals [10,11,12]. The CGF/P microstructure showed discontinuous yielding followed by a lower LYS and UTS but a higher TEL and UEL, and a LYS/UTS ratio much lower than 1. The LYS and the UTS of steel A were plotted for every temperature in each microstructure in Figure 4. Since these LYS vs. T or UTS vs. T curves were quasi-parallel to each other, the difference in the LYS (ΔLYS) or the difference in the UTS (ΔUTS) showed little change with the change in temperature. For example, the ΔLYS was 564 MPa at room temperature and 563 MPa at 210 K, and the ΔUTS was 386 MPa at room temperature and 382 MPa at 210 K, when changing the CGF/P microstructure to the UGF/C microstructure in steel A. Therefore, the influence of the microstructural change on the strength (including the LYS and the UTS) only relied on the microstructure itself but not on the temperature. The flow stress contains two components: (i) thermal stress and (ii) athermal stress [13,14]. Therefore, the microstructural change from the CGF/P microstructure to the UGF/C microstructure only influenced the athermal component of the LYS and the UTS. This microstructural change was mainly attributed to the change of the second phase particles (i.e., from pearlitic lamellae to cementite particles) and ferrite grain size. As a result, the changed stress for dislocation looping around the second phase particles or dislocation emission from sources in neighboring grains or grain boundaries was attributed to the change of the grain size and/or the second phase particles, and hence the microstructure change led to a changed athermal component of the LYS and the UTS. The thermal component of the LYS and the UTS can be studied by the difference in the LYS (ΔLYS) between the LYS vs. T curves or the difference in the UTS (ΔUTS) between the UTS vs. T curves. Since these LYS vs. T curves or UTS vs. T curves were quasi-parallel to each other, the ΔLYS or the ΔUTS caused little change with the change in the microstructure. For example, ΔLYS was 58 MPa for the CGF/P microstructure and 61 MPa for the UGF/C microstructure, and ΔUTS was 77 MPa for the CGF/P microstructure and 73 MPa for the UGF/C microstructure, when decreasing the tensile temperature from room temperature to 210 K. Therefore, a microstructural change from the UGF/C microstructure to the CGF/P microstructure caused little change in the thermal component of the LYS and the UTS.

**Figure 5:**
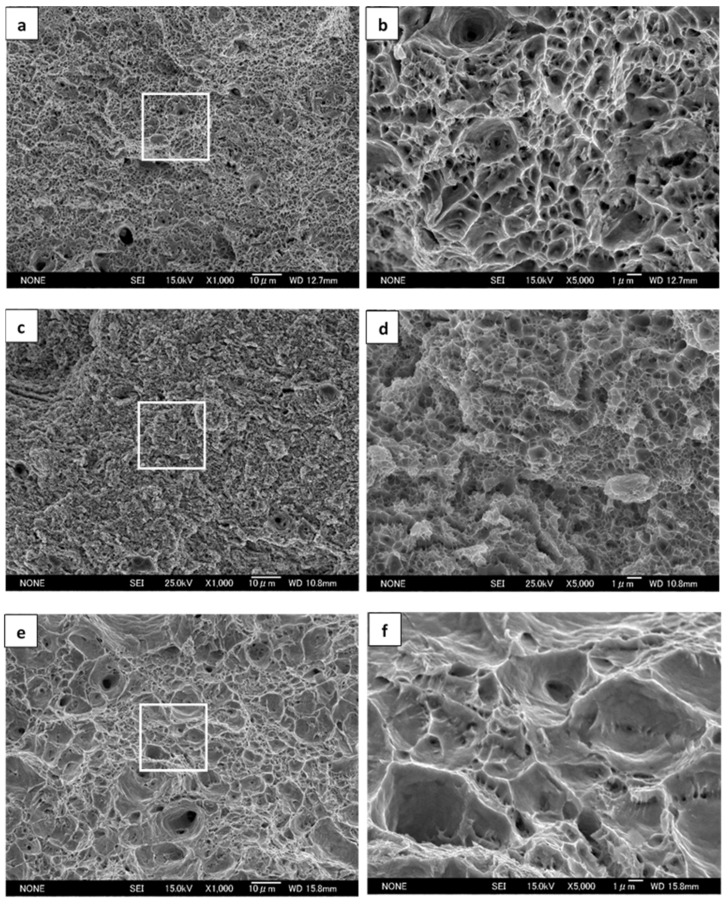
Tensile fracture surfaces tested at room temperature, in which magnified fractographs from the center site are shown within a rectangular box: (**a**,**b**) correspond to the UGF/C microstructure of steel A; (**c**,**d**) correspond to the UGF/C microstructure of steel B; and (**e**,**f**) correspond to the CGF/P microstructure of steel A.

**Figure 6:**
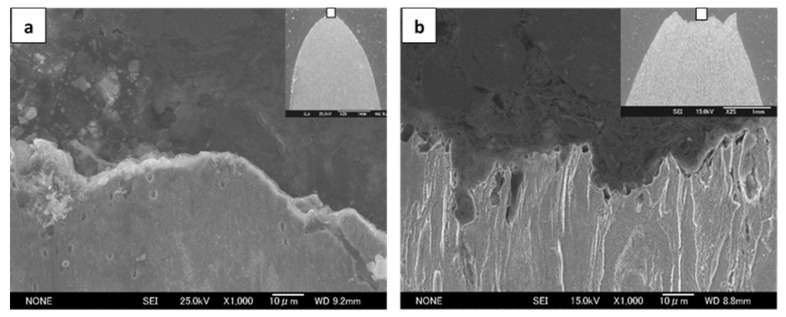
Cross microsections of the sites adjacent to the central fracture surface in steel A shown in Figure 5: (**a**) for the UGF/C microstructure and (**b**) the CGF/P microstructure, showing SEM micrographs with lower magnification embedding on the top right corner and magnified fractographs from the center site within a rectangular box.

Figure 5 shows the SEM tensile fracture morphologies at room temperature, depicting a microvoid coalescence ductile fracture. Figure 6 shows the SEM cross microsections of the sites adjacent to the central tensile fracture surface for the UGF/C microstructure and the CGF/P microstructure of steel A shown in Figure 5, exhibiting dimples of different depths for different microstructures, which substantiates the microvoid nucleation and growth. The UGF/C microstructure and the CGF/P microstructure did not show clear evidence of particle-cracking (i.e., cementite particle-cracking for the UGF/C microstructure and pearlitic lamellae-cracking for the CGF/P microstructure), and hence microvoid nucleation was probably caused predominantly by the decohesion of the particle/matrix bond. The fracture surface of the UGF/C microstructure mainly comprised large amounts of small and closely-spaced dimples accompanied by few slightly-larger widely-spaced dimples. The carbon content increase from 0.15 wt.% (steel A) to 0.45 wt.% (steel B) led to smaller, more closely spaced dimples. The fracture surface of the CGF/C microstructure mainly comprised large, widely spaced dimples accompanied by small, relatively closely spaced dimples. The fracture characteristics reflected the ductility of the corresponding microstructures. A ductile fracture usually involves a plastic unstable localized necking of the intervoid matrix. Some microvoids, which nucleate at the sites of the ferrite matrix, may influence ductile fracture conditions via the localized internal necking of the intervoid matrix because of a more ready formation of the microvoids at the sites of the ferrite matrix than at the sites of the second phase particles (cementite particle or pearlitic lamellae). The microvoids coalesce upon further strain. The strain concentration provides a sufficient plastic strain to nucleate the smaller voids at the positions of second phase particles. Plastic instability will occur soon after these smaller voids form.

**Figure 7:**
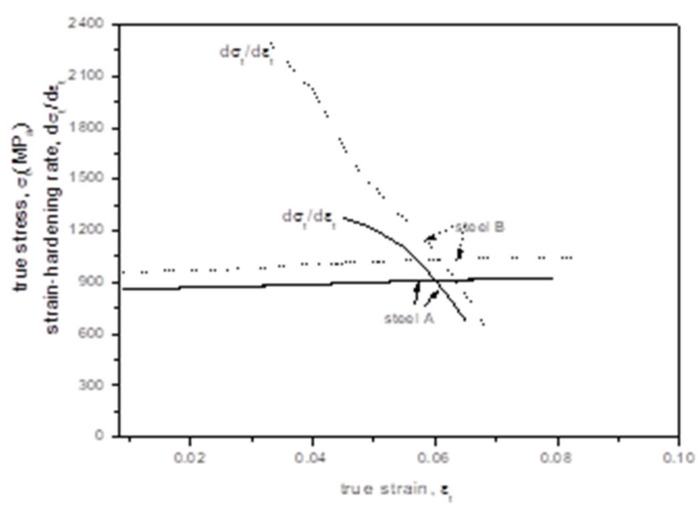
Calculated true stress-strain curves up to the necking occurrence and relation between strain-hardening rate and true strain for the room temperature tensile test.

The LYS and the UTS of steel A increased significantly when the microstructure changed from CGF/P to UGF/C. However, the TEL and the UEL decreased obviously. Such a decrease is a factor restricting the potential applications of the UGF/C microstructure. On the other hand, when the carbon content was increased from 0.15 wt.% (steel A) to 0.45 wt.% (steel B), the strength was higher, but interestingly the ductile (elongation) also increased (Figure 3). Figure 7 shows (i) the calculated true stress-strain curves up to the necking occurrence and (ii) the relation between the strain-harden rate and the true strain, which were obtained from the room temperature tensile tests shown in Figure 3. Obviously, the carbon content increase from 0.15 wt.% to 0.45 wt.% caused an increase in the strain-hardening rate (*dσ*/*dε*). The necking condition in tension is decided by the formula *σ* ≥ *dσ*/*dε*, where *σ* is the flow stress and *ε* is the true strain [15,16]. The uniform elongation is decided by the flow stress and strain-hardening rate. A higher strain-hardening rate increases the uniform elongation. As a result, the uniform elongation increased when the carbon content was increased from 0.15 wt.% to 0.45 wt.%. This provides a prospect for developing plain C–Mn steels with a UGF/C microstructure with a higher carbon content, which are expected to have a higher strength without losing ductility.

## 4. Conclusions

1. The low yield and the ultimate tensile strengths were largely increased when the microstructure was changed from the CGF/P to the UGF/C microstructures, but the total elongation and the uniform elongation were decreased obviously.
2. A microstructural change from the CGF/P microstructure to the UGF/C microstructure had an influence on the athermal component of the lower yield and the ultimate tensile strengths but not on their thermal component.
3. The fracture surface of the UGF/C microstructure mainly contained large amounts of small and closely-spaced dimples, while that of the CGF/P microstructure mainly contained large and widely-spaced dimples. The fracture characteristics reflected the ductility of the corresponding microstructures well.
4. The UGF/C microstructure with a higher carbon content provided a higher strength without a loss of ductility because cementite particles restrained necking.

## Data Availability

Data sharing not applicable.
